# Interleukin-6 Signaling Effects on Ischemic Stroke and Other Cardiovascular Outcomes: A Mendelian Randomization Study

**DOI:** 10.1161/CIRCGEN.119.002872

**Published:** 2020-05-12

**Authors:** Marios K. Georgakis, Rainer Malik, Dipender Gill, Nora Franceschini, Cathie L. M. Sudlow, INVENT Consortium, CHARGE Inflammation Working Group, Martin Dichgans

**Affiliations:** 1 Institute for Stroke and Dementia Research (ISD) (M.K.G., R.M., M.D.), University Hospital, Ludwig-Maximilians-University LMU, Munich, Germany.; 2 Graduate School for Systemic Neurosciences (GSN) (M.K.G.), University Hospital, Ludwig-Maximilians-University LMU, Munich, Germany.; 3 Department of Epidemiology and Biostatistics, School of Public Health, Imperial College London, United Kingdom (D.G., C.L.M.S.).; 4 Department of Epidemiology, UNC Gillings Global School of Public Health, Chapel Hill, NC (N.F.).; 5 Institute for Genetics and Molecular Medicine, University of Edinburgh, United Kingdom (C.L.M.S.).; 6 Munich Cluster for Systems Neurology (SyNergy), Germany (M.D.).; 7 German Centre for Neurodegenerative Diseases (DZNE), Munich, Germany (M.D.).

**Keywords:** cardiovascular diseases, coronary artery disease, genetics, inflammation, stroke

Downregulation of IL-6 (interleukin-6) signaling has been proposed as a strategy for lowering cardiovascular risk. Secondary analyses from CANTOS (Canakinumab Anti-Inflammatory Thrombosis Outcomes Study) demonstrated that the therapeutic benefit of IL-1β (interleukin-1β) inhibition on cardiovascular prevention was associated with the reduction of IL-6 levels and that the residual cardiovascular risk was proportional to post-treatment IL-6 levels.^1^ Moreover, Mendelian randomization (MR) analyses showed variation in IL-6R (IL-6 receptor) gene (*IL6R*) to be associated with risk of coronary artery disease.^2^ Thus, directly interfering with IL-6 signaling might lower cardiovascular risk beyond IL-1β inhibition. Whether such an approach would be effective for ischemic stroke and other cardiovascular outcomes (aortic aneurysm, carotid plaque, peripheral artery disease, atrial fibrillation, heart failure, and thrombotic phenotypes) remains unknown.

Here, we identified genetic proxies for IL-6R-mediated downregulation of IL-6 signaling as variants within a region 300 kB 5′ to 300 kB 3′ to *IL6R* that were associated with lower CRP (C-reactive protein) levels. CRP is a well-established downstream molecule of IL-6 signaling and a clinically useful biomarker for assessing residual inflammatory cardiovascular risk. Variants were derived from a genome-wide association study of 204 402 European individuals (*P*<5×10^−8^; clumped at *r*^*2*^<0.1). We identified 7 single- nucleotide polymorphisms (SNPs) that served as instruments for downregulated IL-6 signaling (3 situated within *IL6R*). In conditional Genome-wide Complex Trait Analysis - conditional and joint (GCTA-CoJo) analyses^3^ adjusting for the lead SNP (rs2228145), *P* values for all SNPs were <0.05 (for SNPs within *IL6R*<0.0083-Bonferroni-corrected threshold) indicating independent effects on CRP levels. F statistics ranged from 81 to 764. To validate these instruments, we explored associations of genetically downregulated IL-6 signaling with circulating upstream regulators (IL-6, soluble IL-6R) and downstream molecules (fibrinogen) of the IL-6 signaling pathway. In accordance with trials testing tocilizumab versus placebo,^2^ genetically downregulated IL-6 signaling was associated with higher circulating IL-6 and soluble IL-6R levels and lower fibrinogen levels (Figure [A]).

**Figure.**
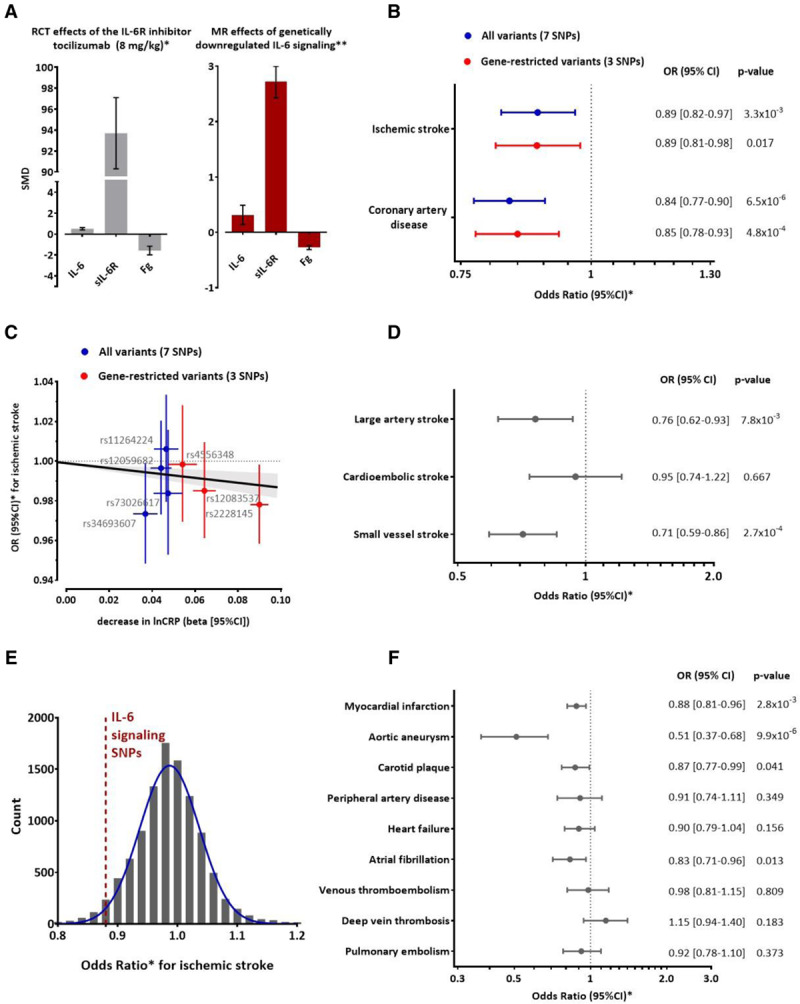
**Genetic proxies for downregulated IL-6 (interleukin-6) signaling and their effects on ischemic stroke and other cardiovascular outcomes in Mendelian randomization analyses (MR).** **A**, Effects of pharmacological inhibition of IL-6R (tocilizumab 8 mg/kg versus placebo for 8–24 weeks) and of genetic downregulation of IL-6 signaling on IL-6 (N_trials_=1446 and N_genetics_=8293), sIL-6R (soluble IL-6 receptor; N_trials_=1465 and N_genetics_=3301), and fibrinogen (Fg) levels (N_trials_=1108 and N_genetics_=120 246). **B**, Genetically downregulated IL-6 signaling in association with ischemic stroke and coronary artery disease as derived from inverse-variance weighted (IVW) analyses using the full set of 7 single-nucleotide polymorphisms (SNPs) as instruments or the 3 SNPs located within the *IL6R* gene. **C**, SNP-specific effects of the associations with ischemic stroke. **D**, Genetically downregulated IL-6 signaling and ischemic stroke subtypes. **E**, Distributions of the effects of 7 randomly selected CRP (C-reactive protein)-decreasing SNPs on risk of ischemic stroke and the position of the effect of the IL-6 signaling downregulating SNPs included in our analyses. **F**, Genetically downregulated IL-6 signaling with other cardiovascular outcomes. Effect sizes for genetically downregulated IL-6 signaling are scaled to the CRP-decreasing effects of tocilizumab (8 mg/kg). Statistical significance thresholds are set at *P*<0.05/3=0.017 for the 3 ischemic stroke subtypes, and at *P*<0.05/9=0.0055 for the 9 cardiovascular outcomes. Associations showing *P* values <0.05 are considered suggestive. SMD indicates standardized mean difference.

Two-sample inverse-variance weighted MR analyses showed genetically downregulated IL-6 signaling to be associated with lower risks of ischemic stroke (MEGASTROKE: 34 217 cases and 404 630 controls) and coronary artery disease (Coronary Artery Disease Genome-wide Replication and Meta-analysis plus Coronary Artery Disease Genetics [CARDIoGRAMplusC4D]: 60 801 cases and 123 504 controls; Figure [B and C]). We further found associations with lower risks of large artery and small vessel stroke, but not cardioembolic stroke (Figure [D]). Alternative MR approaches (weighted median, contamination-mixture, MR-PRESSO) and sensitivity analyses restricted to the variants within *IL6R* all showed consistent association estimates.

MR analyses revealed no significant associations between genetically determined CRP and ischemic stroke or its subtypes independently of whether we used all variants associated with CRP (187 SNPs) or SNPs at the *CRP* locus (24 SNPs). Furthermore, in permutations of MR analyses^4^ randomly selecting 7 of the 187 SNPs associated with CRP, the effects of the 7 SNPs selected as instruments for downregulated IL-6 signaling on ischemic stroke and its subtypes were consistently located below the lowest fifth percentile of the respective distributions (Figure [E]). Thus, the observed effects were independent of the effects of CRP.

Finally, we expanded the analyses to other cardiovascular outcomes in the UK Biobank (321 406 individuals) and phenotype-specific genome-wide association study data sets. Genetically downregulated IL-6 signaling was significantly associated with lower risks of myocardial infarction and aortic aneurysm. We further found suggestive associations (*P*<0.05) with atrial fibrillation and carotid plaque (Figure [F]). Again, these associations were independent of CRP levels.

Our results strongly support the candidacy of IL-6 signaling as a target for vascular prevention over and beyond previous data. CANTOS targeted IL-1β rather than IL-6R thus providing only indirect evidence for a benefit of interfering with IL-6 signaling.^1^ Also, CANTOS explored a combined end point rather than individual cardiovascular outcomes. Regarding stroke, there was a 7% reduction in incident events in the IL-1β arm, which did not reach statistical significance, and data on stroke subtypes were not available.^5^ Our MR results provide evidence for directionally consistent effects of IL-6 signaling on ischemic stroke and other cardiovascular outcomes and offer a solid basis for future trials exploring the benefit of pharmacological IL-6R inhibition for these phenotypes.

Our results are in broad agreement with a recent MR study using the same data sources but a different approach to explore the effects of IL-6 signaling on cardiovascular outcomes.^6^ While that study used plasma levels of soluble IL-6R to proxy the effects of IL-6 signaling, we used CRP levels, which might explain some discrepancies in the results. We did not select variants based on their effects on IL-6 or soluble IL-6R because they are upstream regulators of IL-6 signaling and variants increasing their levels could also upregulate the pathway. Still, IL-6 signaling is complex with a classical and a trans-signaling component and disentangling the 2 subpathways goes beyond the limitations of MR.

In conclusion, this study provides evidence for a causal effect of IL-6 signaling on ischemic stroke, particularly large artery and small vessel stroke, as well as a range of cardiovascular phenotypes. IL-6R blockade might represent a valid therapeutic target for lowering cardiovascular risk and should thus be further investigated in clinical trials.

All data related to the effects of these variants on specific outcomes are publicly available as summary statistics from the respective sources. Data for outcomes derived from the UK Biobank are available after submission of a research proposal. All the data are also available from the corresponding author upon reasonable request. All individual studies had obtained ethical approval by the appropriate institutional review committees, as described in the original publications.

## Acknowledgments

We thank the following consortia for making data publicly available: MEGASTROKE Consortium, CARDIoGRAMplusC4D Consortium, CHARGE Consortium (Coronary Artery Disease Genome-wide Replication and Meta-analysis (CARDIOGRAM) plus the Coronary Artery Disease (C4D) Genetics), AFGen Consortium (Atrial Fibrillation Genetics), the YFS/FINRISK studies (Young Finnish Study/Finland Cardiovascular Risk), and the INTERVAL study. This research has been conducted using the UK Biobank Resource (UK Biobank application 2532, UK Biobank stroke study: developing an in-depth understanding of the determinants of stroke and its subtypes).

## Sources of Funding

Dr Georgakis was funded by scholarships from the Onassis Foundation and the German Academic Exchange Service (DAAD). Dr Gill is supported by the Wellcome Trust 4i Programme (203928/Z/16/Z) and British Heart Foundation Centre of Research Excellence (RE/18/4/34215) at Imperial College London. This project has received funding from the European Union’s Horizon 2020 research and innovation programme (No 666881), SVDs@target (to Dr Dichgans) and No 667375, CoSTREAM (to Dr Dichgans); the DFG as part of the Munich Cluster for Systems Neurology (EXC 2145 SyNergy: ID 390857198) and the CRC 1123 (B3) (to Dr Dichgans); the Corona Foundation (to Dr Dichgans); the Fondation Leducq (Transatlantic Network of Excellence on Pathogenesis of Small Vessel Disease of the Brain, to Dr Dichgans); the e:Med program (e:AtheroSysMed, to Dr Dichgans) and the FP7/2007-2103 European Union project CVgenes@target (grant agreement number Health-F2-2013-601456, to Dr Dichgans).

## Disclosures

None.

## Appendix

### INVENT Consortium members

Sara Lindstrom, Lu Wang, Erin N. Smith, William Gordon, Astrid van Hylckama Vlieg, Mariza de Andrade, Jennifer A. Brody, Jack W. Pattee, Jeffrey Haessler, Ben M. Brumpton, Daniel I. Chasman, Pierre Suchon, Ming-Huei Chen, Constance Turman, Marine Germain, Kerri L. Wiggins, James MacDonald, Sigrid K. Braekkan, Sebastian M. Armasu, Nathan Pankratz, Rabecca D. Jackson, Jonas B. Nielsen, Franco Giulianini, Marja K. Puurunen, Manal Ibrahim, Susan R. Heckbert, Scott M. Damrauer, Pradeep Natarajan, Derek Klarin, Paul S. de Vries, Maria SabaterLleal, Jennifer E. Huffman, Theo K. Bammler, Kelly A. Frazer, Bryan M. McCauley, Kent Taylor, James S. Pankow, Alexander P. Reiner, Maiken E. Gabrielsen, Jean-François Deleuze, Chris J. O’Donnell, Jihye Kim, Barbara McKnight, Peter Kraft, JohnBjarne Hansen, Frits R. Rosendaal, John A. Heit, Bruce M. Psaty, Weihong Tang, Charles Kooperberg, Kristian Hveem, Paul M. Ridker, Pierre Emmanuel Morange, Andrew D. Johnson, Christopher Kabrhel, David-Alexandre Trégouët, Nicholas L. Smith.

### CHARGE Inflammation Working Group

Emelia Benjamin, Daniel I. Chasman, Abbas Dehghan, Tarunveer Singh Ahluwalia, James Meigs, Russell Tracy, Behrooz Z. Alizadeh, Symen Ligthart, Josh Bis, Gudny Eiriksdottir, Nathan Pankratz, Myron Gross, Alex Rainer, Harold Snieder, James G. Wilson, Bruce M. Psaty, Josee Dupuis, Bram Prins, Urmo Vaso, Maria Stathopoulou, Lude Franke, Terho Lehtimaki, Wolfgang Koenig, Yalda Jamshidi, Sophie Siest, Ali Abbasi, Andre G. Uitterlinden, Mohammadreza Abdollahi, Renate Schnabel, Ursula M. Schick, Ilja M. Nolte, Aldi Kraja, Yi-Hsiang Hsu, Daniel S. Tylee, Alyson Zwicker, Rudolf Uher, George Davey-Smith, Alanna C. Morrison, Andrew Hicks, Cornelia M. van Duijn, Cavin Ward-Caviness, Eric Boerwinkle, J. Rotter, Ken Rice, Leslie Lange, Markus Perola, Eco de Geus, Andrew P. Morris, Kari Matti Makela, David Stacey, Johan Eriksson, Tim M. Frayling, Eline P. Slagboom.
